# Cincho-Quinine

**Published:** 1869-06

**Authors:** 


Cincho-Quinine.—We have received a bottle of Cincho-Quinine from the manufacturers, James R. Nichols & Co. of Boston, and have tested its powers, to some extent, in the treatment of intermittent and remittent disease. It is said to possess all the medicinal properties of the bark, and thus have decided advantages over sulphate of quinia. It has very similar appearance, does not seem to be quite so bitter to the taste. The dose is the same, and so far as we have had opportunity to test its efficacy, its effects are the same.

